# The burden of disease among Brazilian older adults and the challenge for health policies: results of the Global Burden of Disease Study 2017

**DOI:** 10.1186/s12963-020-00206-3

**Published:** 2020-09-30

**Authors:** Valéria Maria de Azeredo Passos, Ana Paula Silva Champs, Renato Teixeira, Maria Fernanda Furtado Lima-Costa, Renata Kirkwood, Renato Veras, Bruno Ramos Nascimento, Ana Maria Nogales, Maria Inês Schmidt, Bruce Bartholow Duncan, Ewerton Cousin, Mohsen Naghavi, Fatima Marinho Souza

**Affiliations:** 1 Faculdade de Ciências Médicas de Minas Gerais, Belo Horizonte, Brazil; 2 Faculdade de Medicina, Universidade Federal de Minas Gerais, Belo Horizonte, Brazil; 3 Hospital Sarah, Belo Horizonte, Brazil; 4 Postgraduate Program on Public Health, Universidade Federal de Minas Gerais, Belo Horizonte, Brazil; 5 Fundação Oswaldo Cruz, Belo Horizonte, Brazil; 6 School of Physical Education, Physical Therapy and Occupational Therapy, Universidade Federal de Minas Gerais, Belo Horizonte, Brazil; 7 Universidade Estadual do Rio de Janeiro, Rio de Janeiro, Brazil; 8 Hospital das Clínicas da Universidade Federal de Minas Gerais, Belo Horizonte, Brazil; 9 Universidade de Brasília, Brasília, Brazil; 10 Postgraduate Program in Epidemiology, Universidade Federal do Rio Grande do Sul, Porto Alegre, Brazil; 11 Institute for Health Metrics and Evaluation, University of Washington, Washington, USA

**Keywords:** Older adults, Burden of disease, Life expectancy, Mortality, DALY, Brazil

## Abstract

**Background:**

Brazil is the world’s fifth most populous nation, and is currently experimenting a fast demographic aging process in a context of scarce resources and social inequalities. To understand the health profile of older adults in Brazil is fundamental for planning public policies.

**Methods:**

The estimates were derived from data obtained through the collaboration between the Brazilian Ministry of Health and the Institute of Health Metrics and Evaluation of the University of Washington. The Brazilian Institute of Geography and Statistics provided the population estimates. Data on causes of death came from the Mortality Information System. To calculate morbidity, population-based studies on the prevalence of diseases in Brazil were comprehensively searched, in addition to information obtained from national databases such as the Hospital Information System, the Outpatient Information System, and the Injury Information System. We presented the Global Burden of Disease (GBD) 2017 estimates among Brazilian older adults (60+ years old) for life expectancy at birth (LE), healthy life expectancy (HALE), cause-specific mortality, years of life lost (YLLs), years lived with disability (YLDs), and disability-adjusted life years (DALYs), from 2000 to 2017.

**Results:**

LE at birth significantly increased from 71.3 years (95% UI to 70.9-71.8) to 75.2 years (95% UI 74.7-75.7). There was a trend of increasing HALE, from 62.2 years (95% UI 59.54-64.5) to 65.5 years (95% UI 62.6-68.0). The proportion of DALYs among older adults increased from 7.3 to 10.3%. Chronic noncommunicable diseases are the leading cause of death among middle aged and older adults, while Alzheimer’s disease is a leading cause only among older adults. Mood disorders, musculoskeletal pain, and hearing or vision losses are among the leading causes of disability.

**Conclusions:**

The increase in LE and the decrease of the DALYs rates are probably results of the improvement of social conditions and health policies. However, the smaller increase of HALE than LE means that despite living more, people spend a substantial time of their old age with disability and illness. Preventable or potentially controllable diseases are responsible for most of the burden of disease among Brazilian older adults. Health investments are necessary to obtain longevity with quality of life in Brazil.

## Background

Brazil is the world’s fifth most populous nation, and is currently experimenting a fast demographic aging process in a context of scarce resources and great social inequalities. Since 1950, the shifting age structure showed marked regional differences [1]. While the Southeast, South, and Midwest regions presented a clear demographic transition toward aging, the North and Northeast regions presented elevated mortality and fertility rates and a higher percentage of young people [1]. Along with aging, the concomitant epidemiologic transition increased the incidence of non-communicable diseases (NCDs). Since NCDs are more frequent among older adults, their health tends to be substantially worse, particularly among the poorest populations [2].

While it took a century for the proportion of older adults to increase from 7 to 14% in the population of the developed countries, like France, this same demographic charge is expected to occur in Brazil between 2011 and 2031. This fast aging process increases the financial pressure on health and welfare [3]. By 2020, the excess of economically active population in relation to the dependent population will represent a demographic bonus, due to the greater availability of human resources in the workforce in the last decades. On the other hand, if there is no economic growth, the proportion of the unemployed could jeopardize these demographic opportunities [4]. In the near future, we are about to enter another demographic context, with a larger number of dependent older adults. The amount of health expenditures will depend essentially on the burden of disease, which can be reduced by investments in the prevention and treatment of people throughout their lives, not only in late life [3, 4].

The current Brazilian Constitution has built the basis for public health financing in Brazil. Since 1988, investments in health promotion and prevention, as well in primary care, increased substantially [5]. From 1990 to 2016, Brazil experienced a marked decrease in total mortality and under-five mortality, as well as a reduction in mortality due to communicable diseases and a significant reduction in preventable causes of death. Nevertheless, these improvements were insufficient to eliminate health inequities. States in South and Southeast regions have advanced to later stages of the epidemiological transition toward noncommunicable diseases, compared with states in the North and Northeast regions that continue to face a double burden of communicable and non-communicable diseases, alongside a growing burden due to injuries across the country [6]. The continuance of health achievements and the remediation of inequalities depends on adequate and continuous investment. The Constitutional Amendment No. 95 of May 2017, which prevents an increase in investments in health and education for the next 20 years, may affect the public health system of the country [7].

For the establishment of investment priorities, it is fundamental to understand the health estimates for older adults in the different scenarios of the country. The Global Burden of Disease (GBD) study represents a new paradigm in the evaluation of health trends among the countries. The standardized methodology allows the comparison between localities, and in time [6, 8]. In addition to assessing mortality, it is possible to measure the burden of disability linked to diseases, a fundamental aspect of health, especially among older adults. This article aims to describe the burden of disease for the Brazilian older adults from 2000 to 2017, for the country and states of the Federation.

## Methods

This paper describes the burden of disease for the Brazilian older adults, those aged 60 years or more. In 2010, Brazil had about 207.7 million inhabitants, living in five regions, 26 states, and the Federal District. The number of people aged 60 or more years was estimated in 20,590,597, about 10% of the total population [8]. In order to express the diversity among the five regions of the country, we presented the health metrics of the states with the most numerous elderly population within each region. These are São Paulo (4.771.822 older adults, 11.6% of population) in the Southeast (SE) region, Bahia (1.450.007 older adults, 8.2%) in the Northeast (NE) region, Rio Grande do Sul (1.461.480 older adults, 13.7%) in the South (S) region, Pará (534.461 older adults, 7.1%) in the North (N) region, and Goiás (560.451 older adults, 9.4%) in the Central-West (CW) region. Some states have a higher proportion of older adults, such as Rio de Janeiro (SE, 13%), Paraíba (NE, 12%), and Mato Grosso do Sul (CW, 9.8%), but with a lower absolute number [9].

All estimates, as well as the figures and graphics, were obtained from the Global Burden of Disease 2017, available on the public website of the Institute of Health Metrics and Evaluation (IHME) of the University of Washington. Data points were obtained through the collaboration of the Brazil Ministry of Health and IHME [8]. The graphics and figures were extracted from the IHME site, with the elderly designated as in developed countries, 65+ years old. Since Brazilian legislation classifies as elderly those with 60+ years old, we decided to show the data considering this age range. The Brazilian Institute of Geography and Statistics (IBGE) provided the population estimates based on projections from the 2010 census [9]. Data on causes of death came from the Mortality Information System (SIM) of the Ministry of Health. In order to calculate the disease prevalence and injury incidence, population-based studies on the prevalence of diseases in Brazil were comprehensively searched, in addition to information obtained from national databases of morbidity, such as the Hospital Information System (SIH), the Outpatient Information System (SIA), and the Injury Information System (SINAN) [6, 10].

Mortality estimates were corrected for underreporting and garbage codes. In addition to absolute numbers of deaths and age-standardized mortality, the rates of years of life lost (YLLs) expressed the effect of premature deaths by age, sex, year, and place. YLLs were obtained by multiplying the number of deaths caused by a disease, in each age group, by the remaining life expectancy at this age, regardless of gender [11, 12]. The estimates on mortality, age-standardized mortality rates, and causes of death are available at https://vizhub.healthdata.org/cod/ [13].

The methods to obtain LE (life expectancy) at birth or any age have been previously reported [11]. Healthy life expectancy (HALE) summarizes overall population health, accounting for length of life, and level of health loss by age using years of life lived with disability (YLDs) estimates and the GBD life tables, as previously described [12].

The metric YLDs represents morbidity by multiplying the prevalence of each disease-related sequelae by its disability weight [14, 15]. A specific software, DisMod-MR, was used for data processing on Bayesian meta-regression models to generate consistent estimates of incidence, prevalence, duration of disease remission, and excess risk of death for each disease [14, 15]. The sources of data used are available at: https:/ghdx.healthdata.org/gbd-2017/data-input-sources [16].

Estimates of disability-adjusted life years (DALYs) lost were obtained by adding YLLs and YLDs, the burden of disease being a sum of lethal and non-lethal diseases [14]. In this study, the distributions of mortality by the main causes of death and the distribution by DALYs were very similar, given the greater impact of YLLs in this age group (data not shown). Therefore, we will present the main causes of death and the YLDs by place, sex, and age groups: 60-64, 65-69, 70-74, 75-79, and 80+ years.

All estimates were drawn 1000 times, and the 95% uncertainty limits value were defined by 2.5° and 97.5° of the estimated values. The 95% uncertainty intervals (95% UI) include uncertainties of all sources and modeling steps, such as sample size variability of the various sources of data, adjustments to general mortality sources, parameter uncertainty in model estimation, specification of uncertainty for models of causes of death, and different data availability by age, sex, year, and location [10].

The analysis of causes of death or disability comprises different degrees of disaggregation. Level 1 divides diseases into three broad groups (1—communicable diseases, maternal, and nutritional diseases, 2—non-communicable diseases, and 3—injuries). Level 2 contains 21 groups of diseases, such as cardiovascular diseases, cancers, and traffic accidents. Level 3 shows separate causes for 168 diseases, such as chronic renal failure. Level 4, on the other hand, breaks down diseases into further 289, for example, chronic renal failure due to diabetes, and level 5 describes diseases with degrees of severity (879 diseases and their sequelae) [10]. In this study, we use level 1 to compare the metrics between the older adults and the younger population. The burden of diseases was shown at level 4, since more aggregated levels did not express well the differences in disease burden between 2000 and 2017 (data not shown).

The socio-demographic index (SDI) is a composite measure that aggregates the total fertility rate under the age of 25 years, the lag distributed income per capita, and the average educational attainment of each location [11]. The scores range from zero to one, that is, the lowest income, lowest education, and highest fertility, to the highest income, highest education, and lowest fecundity. According to the value of the SDI, the sites are classified as high, medium high, medium, medium low, or low SDI. Overall, Brazil ranked in the medium SDI category in 2015 [6, 10].

## Results

In Brazil, between 2000 and 2017, for both sexes, life expectancy at birth increased approximately 4 years, from 71.4 years (95% to 71.1-71.7) to 75.5 years (95% UI 75.3-75.7). HALE increased from 61.7 years (95% UI 59.0-64.1) to 65.4 years (95% UI 62.6-68.0). These estimates were very similar among the states, taking into account the 95% UI values (Table 1).

**Table 1:** Life expectancy (LE) and healthy life expectancy (HALE) to population of Brazil and selected states, in 2000 and 2017. Estimates from GBD study 2017

Local	Social demographic index	LE at birth (95% UI)	HALE at birth (*N* (95% UI))	LE at 60 years old (*N* (95% UI))	HALE at 60 years old (*N* (95% UI))
**2000**					
**Brazil**	0.562	71.4 (71.1-71.7)	61.7 (64.1-59)	20.6 (20.6-20.6)	15.7 (16.9-14.4)
São Paulo (SE)	0.603	71 (70.9-71.1)	61.5 (63.8-58.8)	19.6 (19.5-19.6)	15 (16.1-13.7)
Rio Grande do Sul (S)	0.630	72.4 (72.3-72.6)	62.3 (64.8-59.6)	19.7 (19.7-19.8)	15 (16.1-13.7)
Pará (N)	0.473	73.1 (72.5-73.8)	62.9 (65.5-60)	22.2 (22-22.5)	17 (18.3-15.5)
Bahia (NE)	0.475	71.7 (70.9-72.6)	61.9 (64.4-59)	22.2 (21.8-22.6)	17 (18.3-15.5)
Goiás (CW)	0.538	73.1 (73-73.3)	63.2 (65.6-60.3)	20.6 (20.5-20.6)	15.7 (16.9-14.4)
**2017**					
**Brazil**	0.663	75.5 (75.3-75.7)	65.4 (67.8-62.5)	22.1 (22.1-22.2)	17 (18.3-15.6)
São Paulo (SE)	0.693	76.1 (75.8-76.3)	65.9 (68.3-63)	21.8 (21.7-22)	16.7 (18-15.3)
Rio Grande do Sul (S)	0.720	75.4 (75.1-75.6)	65 (67.5-62.1)	21.7 (21.5-21.8)	16.6 (17.8-15.2)
Pará (N)	0.579	75.5 (75.2-75.9)	65.4 (67.9-62.6)	22.5 (22.3-22.7)	17.3 (18.6-15.9)
Bahia (NE)	0.591	75.5 (75-76)	65.3 (67.8-62.3)	23 (22.8-23.2)	17.7 (19-16.2)
Goiás (CW)	0.650	75.5 (75.1-75.8)	65.3 (67.7-62.3)	23 (22.8-23.2)	17.2 (18.5-15.7)

*SE* southeast region, *S* south region, *N* north region, *NE* northeast region, CW central-west region

The national LE at age 60 increased less than 2 years, from 20.6 years (95% UI 20.6-20.6) to 22.1 years (95% UI 22.1-22.2) between 2000 and 2017. It is noteworthy that about one quarter of this time will be lost to disability, HALE equal to 15.7 years (95% UI 14.4-16.9) in 2000 and 17.0 years (95% II 15.6-18.3) in 2017 (Table 1).

**Fig. 1:**
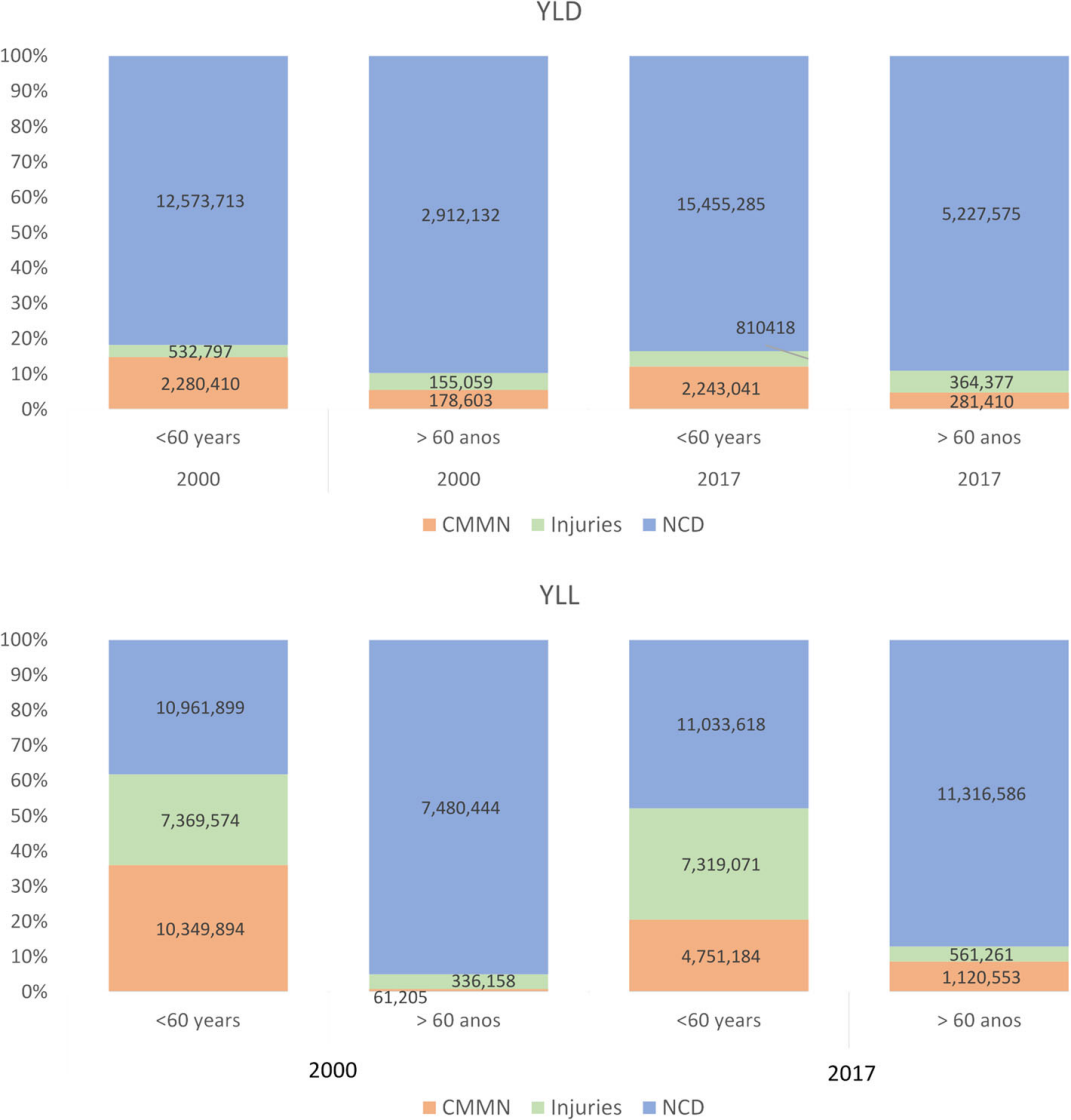
Distribution of the burden of disease per 100,000 inhabitants for Brazil, both sexes and age groups, in 2000 and 2017. YLD, years lost by disability; YLL, years of life lost; DALY, disability-adjusted life years

Age-standardized DALYs for all causes decreased from 35,723.86 (95% UI 32,900.92-39,077.70) in 2000 to 27,894.03 (95% UI 25,164.67-31,031.81) in 2017. In the same period, we noticed a prominent increase not only in the absolute number but also in the proportion of DALYs among the older adults. The burden of disease to all ages and causes was equal to 55,742,743 and 60,487,378 DALYs in 2000 and 2017, with older adults representing 20.9% and 31.2% of the total DALYs for all ages in the period. Although there was a decrease in age-standardized rates, the distribution of the burden of the disease to the population up to 60 years, and the older adults, revealed a higher proportion of all metrics by noncommunicable diseases among the older adults, especially for YLL distribution (Fig. 1).

### Mortality

**Fig. 2:**
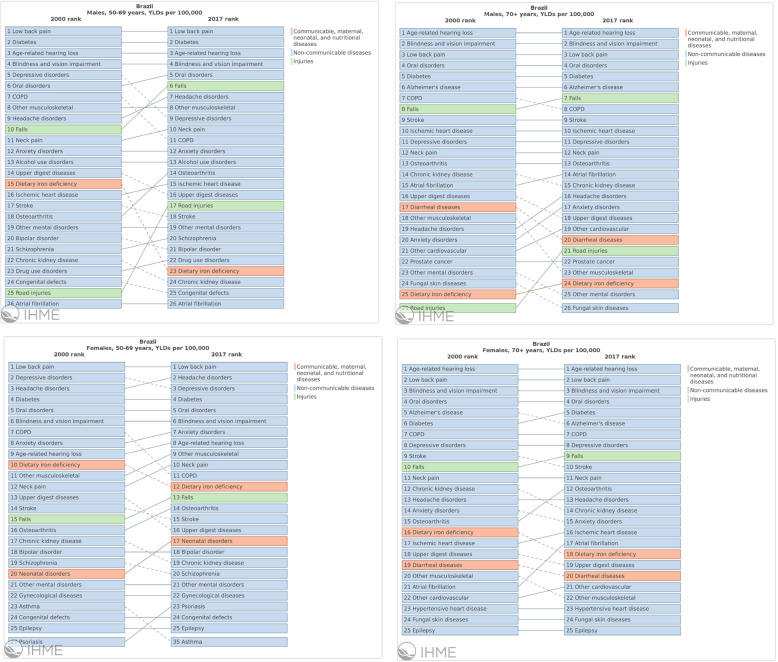
Main causes of deaths in Brazil among middle-aged adults and elderlies, by sex, 2000 and 2017. PC, percent changing

Between 2000 and 2017, despite small changes, most of the ten leading causes of death remained the same among the elderly of both sexes, such as ischemic heart disease, stroke, chronic obstructive pulmonary disease, and diabetes. Breast and colon cancers are important leading cancer causes among women, while prostate and lung cancer predominating among men. Alzheimer’s disease and other dementias emerged as an important cause of death among the elderly (Fig. 2).

External causes of death, such as road injuries and interpersonal violence, remain as leading causes of mortality among the middle aged during the period, especially for men. In 2017, mortality by falls increased for both sexes with aging, being the 13th and 16th causes of death among female and male elderlies, respectively (Fig. 2).

**Fig. 3:**
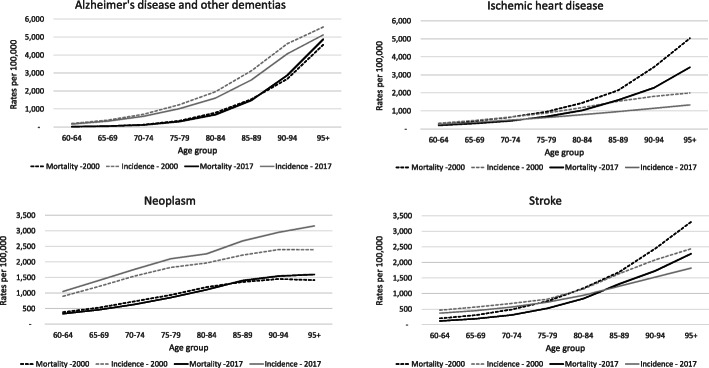
Incidence and mortality of four leading causes of burden of disease among elderlies, in 2000 and 2017

Figure 3 shows the increase of both incidence and mortality with aging for ischemic heart disease, stroke, Alzheimer’s disease, and neoplasms, especially among the oldest old, those with age of 80 or more years old.

In Brazil, ischemic heart disease was the leading cause of death between 2000 and 2017 for both sexes, although there was a decrease in mortality rates from 31.8% for the youngest (60-64 years) to 24.9% for the oldest elderlies (80+ years). There was a clear gradient of increasing mortality with aging, the mortality rate ranging from 299.5/100,000 inhabitants (95% UI 291.8-308.0) and 204.4/100,000 inhabitants (95% UI 196.1-211.3) among those with 60 to 64 years old to 1923.9/100,000 inhabitants (95% UI 1890.4-1970.2) and 1444.7/100,000 inhabitants (95% UI 1403.3-1485.4) among those with 75 to 79 years old, in 2000 and 2017, respectively (Table 2). When we observed the mortality rates in the states, there is a greater amplitude of the 95% UI for the states of Bahia (NE) and Goiás (CW), in the majority of the age groups, in both years. The State of Pará (N) presented the lowest rates of mortality by age in the period, as well as the lowest percentages of decrease. The states of São Paulo (SE) and Rio Grande do Sul (S) presented the highest risk of death due to ischemic heart disease, but also with the highest decreases in the period, around 40% (Table 2).

**Table 2:**
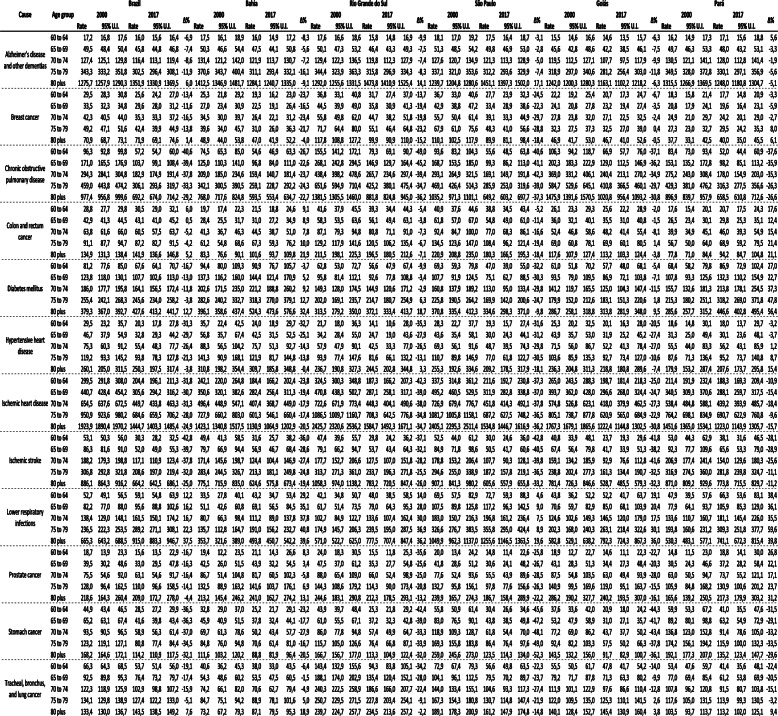
Distribution of death rates per 100,000 inhabitants for Brazil and selected states, both sexes and by age groups, 2000 and 2017

Ischemic stroke was the second leading cause of death among older adults, also presenting a decrease in all age groups. There is a clear aging gradient in both years, the rates among the oldest old adults being almost 18 times greater than the youngest ones. The states from the most developing regions, São Paulo and Rio Grande do Sul, presented the higher mortality rates (Table 2).

Mortality due to diabetes remained relatively stable in the period for both sexes (Fig. 1). We noticed that death rates for all age groups decreased in São Paulo (SE), whereas there was an increase of death rates among the 75+ years old in the states of Rio Grande do Sul (S), Bahia (NE), and Goiás (CW). In Pará (N), the risk of dying due to diabetes increased to all age groups in this period (Table 2).

In this period, we noticed a decrease in trend of deaths by COPD while deaths by lower respiratory infections increased pari passu. The rates of mortality by breast cancer decreased all over the country while deaths by prostate cancer are still increasing in the less developing states of Bahia and Pará (Table 2).

### Disability

**Fig. 4:**
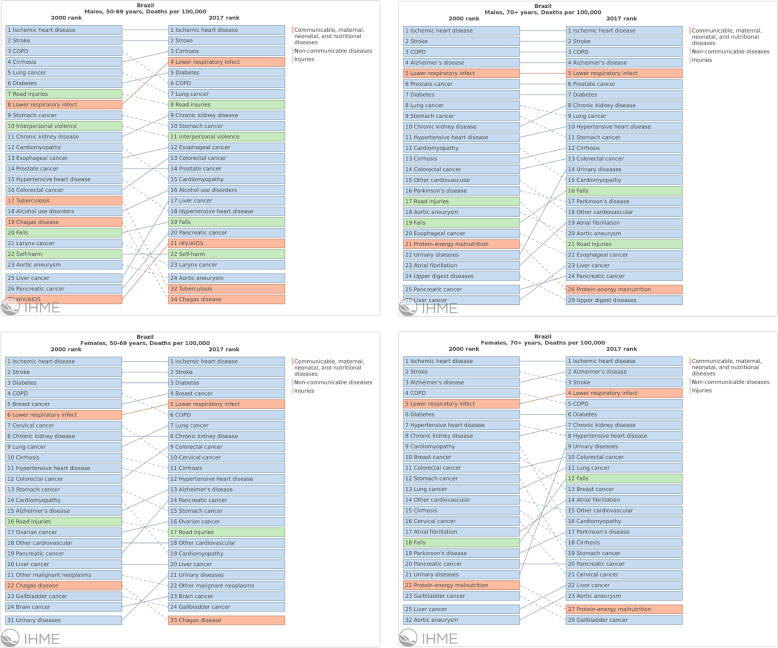
Main causes of disability among middle-aged adults and elderlies, by sex, in 2000 and 2017

From 2000 to 2017, for both middle aged and the elderly, most of the leading causes of incapacity (YLD) remained the same, with minor changes in the rank within the age strata. Low-back pain is the first or second cause of disability among both sexes and all over the period. Among younger women, diseases related to stress are prominent: depressive disorders, headache, and anxiety disorders are among the four leading causes of YLD. Among older adults, for both sexes, low-back pain, age-related hearing loss, blindness, and oral disorders and diabetes are the top five causes of disability. Dietary iron deficiency and diarrheal diseases are declining in the period for both age strata. On the other hand, disability by Alzheimer’s disease and falls increased among the elderly from 2000 to 2017 (Fig. 4).

The burden of disability increases with aging, with the oldest elderly presenting two to three times more YLD for visual and oral disorders, COPD, ischemic heart disease, and falls than 60-64 years olds. The YLD rates for depressive disorders, neck, and low back pain are relatively the same among the age groups, while migraine is the only cause of YLD that decreases with aging.

Between 2000 and 2017, comparing the youngest and oldest old, there was an increase in the national burden due to falls increased from 13.3 to 34.6%. This burden was higher in the developed states of Rio Grande do Sul (20.6-44.6%) and São Paulo (31.4-47.7%) and smaller in the less developed states of Pará (−17.6-10.1%). In the period, we can also notice the decreasing trends for depressive and oral disorders, ischemic heart disease, ischemic stroke, and diabetes mellitus in all the states. The burden for migraine and low back pain are relatively stable in time (Table 3).

**Table 3:** Distribution of YLD rates per 100.000 inhabitants for Brazil and selected States, both sexes and by age groups, 2000 and 2017

Cause	Age group	Brazil							Bahia							Rio Grande do Sul							São Paulo							Goiás							Pará						
		2000			2017			Δ%	2000			2017			Δ%	2000			2017			Δ%	2000			2017			Δ%	2000			2017			Δ%	2000			2017			Δ%
		Rate	95% U.I.		Rate	95% U.I.			Rate	95% U.I.		Rate	95% U.I.			Rate	95% U.I.		Rate	95% U.I.			Rate	95% U.I.		Rate	95% U.I.			Rate	95% U.I.		Rate	95% U.I.			Rate	95% U.I.		Rate	95% U.I.		
Blindness and vision impairment	60 to 64	1004.3	661.5	1509.3	1009.9	654.6	1532.1	0.6	1058.0	685.5	1601.1	1050.3	669.7	1598.0	-0.7	983.8	641.4	1474.0	989.3	639.9	1508.1	0.6	940.6	610.2	1422.2	982.9	642.7	1498.4	4.5	1024.7	661.6	1537.2	1018.5	660.7	1555.3	-0.6	1068.5	694.3	1618.2	1061.2	686.8	1625.4	-0.7
	65 to 69	1275.7	859.0	1881.7	1279.7	854.3	1898.1	0.3	1347.9	908.0	1992.7	1328.4	874.6	2000.3	-1.4	1248.9	840.5	1839.2	1252.1	826.6	1852.5	0.3	1200.2	809.0	1787.5	1249.5	833.9	1848.9	4.1	1299.9	877.5	1930.1	1288.1	863.0	1904.0	-0.9	1348.9	913.3	1977.0	1339.8	885.4	1977.6	-0.7
	70 to 74	1651.6	1131.6	2367.2	1647.9	1127.8	2387.2	-0.2	1743.2	1191.2	2504.8	1709.2	1155.6	2466.2	-2.0	1615.7	1111.6	2320.2	1602.0	1096.4	2321.2	-0.8	1554.3	1061.3	2216.0	1618.4	1113.6	2339.0	4.1	1675.3	1152.2	2414.7	1653.1	1134.2	2391.6	-1.3	1739.4	1208.0	2468.7	1716.2	1172.4	2490.9	-1.3
	75 to 79	2147.5	1494.8	2944.4	2129.4	1472.6	2979.7	-0.8	2254.6	1556.1	3118.0	2199.8	1535.7	3068.5	-2.4	2098.8	1465.3	2938.9	2060.8	1413.3	2856.6	-1.8	2020.7	1401.8	2774.0	2101.7	1452.1	2935.3	4.0	2167.9	1491.0	2967.2	2133.7	1471.0	2952.4	-1.6	2249.5	1584.8	3063.3	2211.4	1502.6	3079.6	-1.7
	80 plus	3239.4	2324.9	4236.6	3210.2	2302.9	4229.6	-0.9	3468.5	2493.5	4589.3	3388.8	2408.5	4472.3	-2.3	3079.1	2173.7	4058.1	3099.5	2216.4	4061.9	0.7	2996.5	2127.0	3971.4	3143.9	2250.3	4206.1	4.9	3097.0	2210.6	4085.4	3153.9	2239.1	4181.6	1.8	3420.1	2455.6	4544.2	3326.8	2356.9	4368.7	-2.7
Chronic obstructive pulmonary disease	60 to 64	3527.8	3315.1	3750.1	2189.5	2031.4	2359.7	-37.9	2776.5	2458.9	3082.4	2008.9	1760.2	2285.2	-27.6	5387.6	4909.8	5907.6	2910.0	2558.3	3265.1	-46.0	3574.0	3227.9	3969.6	2255.8	1983.2	2533.7	-36.9	3871.4	3470.8	4273.4	2480.4	2186.5	2776.3	-35.9	3018.3	2705.4	3336.2	1941.7	1683.9	2204.7	-35.7
	65 to 69	4912.5	4666.9	5181.5	3120.5	2920.0	3341.7	-36.5	3686.9	3292.2	4095.3	2827.3	2512.0	3186.4	-23.3	7375.5	6735.8	8040.2	4244.5	3803.1	4707.1	-42.5	4991.7	4589.0	5472.6	3150.8	2780.1	3523.0	-36.9	5698.0	5186.7	6218.4	3740.5	3321.9	4209.3	-34.4	4329.1	3867.3	4784.0	2874.2	2523.7	3262.1	-33.6
	70 to 74	6546.6	6257.1	6837.8	4271.2	4017.5	4512.1	-34.8	4786.9	4299.3	5311.8	3683.2	3267.9	4125.7	-23.1	9465.9	8717.3	10271.9	5955.9	5367.9	6544.5	-37.1	6668.1	6070.0	7281.2	4159.9	3721.6	4624.7	-37.6	7997.4	7293.2	8711.9	5388.9	4856.9	5984.0	-32.6	6020.2	5436.5	6644.4	4042.9	3601.7	4527.3	-32.8
	75 to 79	7798.3	7461.6	8141.2	5432.1	5154.6	5729.1	-30.3	5926.8	5303.0	6629.0	4583.4	4093.7	5115.0	-22.7	10828.7	9974.8	11674.2	7304.3	6620.2	8062.2	-32.5	8109.2	7411.0	8824.4	5305.9	4799.9	5870.5	-34.6	9684.9	8871.5	10544.6	6993.5	6334.8	7705.0	-27.8	7205.9	6493.8	7958.3	5428.8	4825.9	6028.8	-24.7
	80 plus	9531.8	9172.2	9853.4	6877.6	6572.4	7217.0	-27.8	7193.3	6684.4	7720.5	5694.3	5288.4	6129.7	-20.8	13470.8	12635.4	14257.3	8699.0	8093.5	9312.5	-35.4	10470.5	9758.6	11131.3	6834.5	6318.1	7328.5	-34.7	13864.6	13025.0	14788.3	9688.9	9074.9	10383.9	-30.1	8503.5	7946.7	9124.1	6398.8	5913.7	6920.0	-24.8
Depressive disorders	60 to 64	1188.5	815.5	1631.5	906.2	629.9	1249.4	-23.8	1029.8	680.4	1456.7	780.0	541.1	1084.7	-24.3	1408.0	937.1	1964.6	1021.6	695.7	1437.5	-27.4	1251.2	864.6	1696.9	909.1	614.3	1267.8	-27.3	1234.1	816.4	1732.5	944.2	635.4	1313.5	-23.5	1000.0	666.7	1428.1	706.0	476.0	1006.2	-29.4
	65 to 69	1126.9	787.5	1528.9	881.4	614.8	1195.3	-21.8	973.9	672.6	1356.1	761.6	529.9	1043.5	-21.8	1338.8	907.4	1846.3	996.6	689.0	1372.0	-25.6	1197.6	853.7	1614.7	885.1	612.1	1221.7	-26.1	1161.9	789.3	1588.9	919.2	625.3	1251.1	-20.9	946.3	654.3	1304.9	700.3	472.7	976.7	-26.0
	70 to 74	1052.6	725.1	1424.7	854.3	594.4	1162.0	-18.8	901.6	614.8	1241.2	742.2	519.9	1010.4	-17.7	1257.0	848.4	1715.5	977.7	664.2	1342.7	-22.2	1131.2	789.1	1519.3	861.5	593.1	1181.7	-23.8	1067.1	721.5	1481.0	887.3	610.2	1216.0	-16.9	887.4	601.7	1231.7	691.3	471.9	950.7	-22.1
	75 to 79	971.2	662.2	1347.9	816.6	559.9	1127.2	-15.9	824.6	553.9	1164.9	714.3	482.3	986.3	-13.4	1167.7	771.4	1626.6	938.0	626.3	1307.6	-19.7	1065.1	740.5	1456.5	823.4	562.0	1136.6	-22.7	978.9	656.1	1379.0	851.2	574.0	1191.0	-13.0	823.1	547.1	1160.1	678.2	448.3	970.8	-17.6
	80 plus	927.5	632.7	1259.9	842.3	582.4	1132.8	-9.2	799.2	538.2	1120.5	738.9	506.0	999.5	-7.6	1104.3	733.2	1517.6	970.8	659.1	1319.7	-12.1	1044.4	725.6	1398.6	875.9	596.8	1169.0	-16.1	913.2	622.4	1272.0	858.5	583.8	1184.5	-6.0	810.8	546.4	1116.3	699.2	472.3	968.1	-13.8
Diabetes mellitus	60 to 64	3446.5	3039.7	3939.3	2944.4	2551.2	3398.6	-14.6	3805.0	3241.5	4438.4	3700.6	3144.5	4359.2	-2.7	2770.7	2346.0	3284.1	2563.2	2144.7	3053.7	-7.5	3198.2	2676.1	3774.4	2306.3	1887.5	2793.3	-27.9	2731.0	2297.0	3220.9	2524.2	2095.5	3005.5	-7.6	2904.6	2455.3	3397.7	3489.7	2958.3	4106.9	20.1
	65 to 69	4130.2	3682.7	4631.8	3649.2	3256.6	4130.5	-11.6	4421.1	3798.8	5094.5	4597.8	3921.0	5417.1	4.0	3321.3	2789.6	3896.3	3220.8	2683.1	3797.4	-3.0	3839.2	3217.0	4523.7	2831.4	2366.1	3325.8	-26.2	3291.6	2791.2	3886.8	3008.3	2558.9	3550.0	-8.6	3552.4	3043.8	4158.1	4216.0	3568.9	4895.7	18.7
	70 to 74	4801.2	4323.8	5321.0	4310.2	3847.0	4845.1	-10.2	5067.2	4334.4	5858.4	5463.9	4674.2	6319.6	7.8	3959.5	3393.9	4570.0	3868.3	3299.8	4551.2	-2.3	4409.5	3752.4	5113.7	3298.6	2807.6	3899.7	-25.2	3847.8	3272.9	4458.2	3415.4	2866.0	4011.9	-11.2	4022.4	3433.8	4637.6	5207.0	4396.5	6052.9	29.4
	75 to 79	5079.9	4587.6	5632.6	4885.7	4392.7	5419.1	-3.8	5408.5	4644.3	6259.7	6012.9	5207.8	7044.8	11.2	4159.1	3547.3	4788.7	4353.0	3644.2	5065.4	4.7	4726.9	4051.7	5470.1	3734.9	3086.7	4414.9	-21.0	3913.8	3320.0	4536.0	3809.3	3179.9	4492.4	-2.7	4282.5	3603.4	4961.8	5885.6	5056.4	6750.0	37.4
	80 plus	4684.3	4212.8	5189.4	4856.1	4393.6	5386.1	3.7	4614.9	4056.3	5235.3	5560.0	4887.2	6288.7	20.5	4086.2	3563.7	4668.8	4392.5	3823.3	4988.9	7.5	4753.8	4157.4	5456.2	4044.2	3497.0	4657.9	-14.9	3913.6	3371.2	4485.4	3864.8	3355.5	4442.5	-1.2	3619.4	3157.6	4126.3	5036.5	4443.9	5680.7	39.2
Edentulism and severe tooth loss	60 to 64	867.0	548.1	1261.4	827.5	525.1	1205.4	-4.6	823.0	517.8	1209.6	792.9	499.3	1177.5	-3.7	781.1	493.1	1148.7	745.8	468.6	1105.9	-4.5	952.2	596.3	1389.3	902.0	563.1	1318.7	-5.3	871.4	553.9	1261.8	848.2	536.6	1244.5	-2.7	807.1	517.6	1175.2	779.2	495.3	1148.7	-3.5
	65 to 69	1219.6	811.2	1733.4	1164.2	767.5	1657.4	-4.5	1170.4	771.5	1664.0	1122.5	730.3	1604.6	-4.1	1117.2	736.0	1589.2	1061.3	693.2	1528.2	-5.0	1324.0	881.1	1872.7	1257.5	820.9	1769.5	-5.0	1228.6	808.9	1740.7	1193.6	786.6	1709.1	-2.8	1146.5	760.9	1619.8	1106.3	725.8	1580.7	-3.5
	70 to 74	1422.7	952.0	1985.8	1367.1	903.7	1931.9	-3.9	1367.4	910.5	1937.5	1323.5	878.5	1895.3	-3.2	1318.5	862.1	1889.0	1262.0	830.4	1800.3	-4.3	1530.8	1027.0	2145.8	1464.7	970.1	2061.3	-4.3	1425.0	955.7	1998.2	1397.6	932.1	1954.7	-1.9	1345.6	899.7	1892.8	1303.5	867.7	1849.1	-3.1
	75 to 79	1467.3	985.6	2037.8	1420.1	947.8	1977.4	-3.2	1406.9	940.2	1940.0	1374.2	916.0	1913.4	-2.3	1370.1	909.4	1921.5	1314.6	862.7	1852.0	-4.1	1579.2	1061.6	2209.9	1516.7	1013.5	2117.9	-4.0	1469.6	992.1	2048.3	1449.0	972.5	2020.8	-1.4	1390.1	944.0	1930.1	1360.9	909.1	1899.7	-2.1
	80 plus	1391.8	937.6	1898.5	1344.2	907.4	1838.9	-3.4	1318.5	882.8	1808.0	1283.4	858.4	1773.4	-2.7	1308.6	874.8	1807.1	1249.7	834.0	1727.8	-4.5	1515.6	1023.0	2090.0	1442.8	965.8	1982.3	-4.8	1394.9	946.5	1908.3	1369.1	914.8	1881.9	-1.8	1312.8	884.0	1798.9	1273.6	858.0	1763.5	-3.0
Falls	60 to 64	792.8	658.7	955.3	898.1	729.5	1098.8	13.3	731.2	603.0	869.2	830.0	677.3	1007.4	13.5	675.7	531.4	833.7	815.2	643.3	1023.2	20.6	1001.0	814.8	1216.7	1042.4	837.7	1277.7	4.1	867.3	689.1	1066.4	1139.6	901.7	1440.9	31.4	824.0	652.4	1016.8	678.6	544.5	836.0	-17.6
	65 to 69	893.6	729.2	1087.5	1058.7	851.7	1297.7	18.5	820.1	663.5	993.4	952.4	768.5	1167.2	16.1	739.2	574.8	926.9	969.3	758.7	1225.9	31.1	1109.5	898.2	1343.8	1244.5	995.2	1529.5	12.2	1023.0	804.6	1273.5	1394.2	1098.7	1734.3	36.3	892.5	707.6	1114.3	804.0	648.2	993.0	-9.9
	70 to 74	1041.4	850.8	1271.0	1278.2	1034.8	1559.2	22.7	938.7	774.4	1138.3	1091.7	880.7	1337.1	16.3	867.0	680.2	1092.1	1186.8	941.3	1490.2	36.9	1251.0	998.1	1552.7	1504.7	1192.1	1839.9	20.3	1239.3	979.9	1526.1	1697.1	1356.6	2091.7	36.9	1039.1	818.2	1279.6	893.6	712.8	1108.7	-14.0
	75 to 79	1358.3	1135.4	1621.2	1742.2	1459.1	2079.7	28.3	1218.5	1005.8	1452.7	1403.9	1149.6	1699.1	15.2	1138.8	905.0	1392.1	1633.8	1333.6	1997.8	43.5	1649.1	1358.4	2001.1	2067.3	1714.2	2493.8	25.4	1572.8	1259.5	1927.1	2315.9	1891.4	2798.9	47.2	1238.5	995.3	1516.6	1210.3	986.6	1466.6	-2.3
	80 plus	2064.2	1741.3	2438.6	2779.5	2357.0	3255.0	34.6	1727.0	1450.5	2058.5	2186.1	1825.4	2580.7	26.6	1872.8	1518.2	2246.4	2707.3	2235.5	3218.2	44.6	2582.2	2148.5	3089.8	3339.7	2793.4	3940.4	29.3	2531.0	2092.2	3013.1	3737.5	3140.0	4386.3	47.7	1686.4	1362.4	2048.6	1856.5	1543.1	2196.7	10.1
Ischemic heart disease	60 to 64	8318.2	8075.9	8582.6	5744.2	5508.9	5952.6	-30.9	6767.8	6145.0	7369.6	5199.1	4699.6	5696.9	-23.2	9007.8	8344.7	9678.8	5300.5	4725.5	5830.3	-41.2	9347.8	8714.9	9997.8	5938.8	5432.7	6465.3	-36.5	7382.4	6805.3	8003.5	5588.6	5108.4	6118.6	-24.3	5936.7	5403.1	6487.9	5309.6	4787.8	5869.8	-10.6
	65 to 69	10241.0	9962.3	10549.3	7192.3	6919.0	7443.5	-29.8	8207.6	7490.6	8977.6	6662.5	6059.0	7312.7	-18.8	10938.6	10174.1	11665.2	6797.7	6120.8	7503.0	-37.9	11475.3	10695.6	12239.2	7337.9	6678.9	7963.1	-36.1	9172.8	8427.2	9925.9	6984.7	6355.8	7623.5	-23.9	7974.8	7233.3	8681.1	6796.3	6160.4	7479.1	-14.8
	70 to 74	12340.2	12000.5	12694.4	8594.4	8249.4	8902.1	-30.4	9449.8	8579.0	10384.5	7812.4	7094.8	8561.5	-17.3	13602.9	12683.3	14531.6	8589.8	7769.0	9350.4	-36.9	13662.9	12798.7	14588.2	8638.0	7915.2	9391.3	-36.8	10881.2	10017.0	11776.7	8011.0	7316.6	8847.7	-26.4	10218.9	9262.1	11124.6	8398.4	7560.2	9262.1	-17.8
	75 to 79	13964.9	13563.7	14415.7	10180.4	9779.9	10560.9	-27.1	10792.4	9801.6	11891.6	8985.2	8209.5	9856.3	-16.7	15896.1	14781.4	16908.5	10545.4	9513.7	11588.4	-33.7	15823.7	14756.9	16920.5	10224.9	9339.2	11056.0	-35.4	11886.2	10942.1	12900.4	9255.8	8435.1	10203.2	-22.1	11315.5	10385.0	12367.8	10266.6	9273.3	11279.6	-9.3
	80 plus	16261.5	15887.4	16734.4	11781.6	11376.6	12153.7	-27.5	11531.9	10859.1	12355.2	9034.8	8465.2	9619.0	-21.7	20828.9	19791.7	21858.0	12929.0	12143.9	13726.6	-37.9	20560.4	19577.2	21532.4	12654.6	11880.5	13428.4	-38.5	15331.9	14471.9	16288.6	10404.7	9721.6	11105.4	-32.1	12031.0	11250.2	12771.4	10215.6	9500.8	10952.9	-15.1
Ischemic stroke	60 to 64	1688.2	1584.9	1792.1	1063.2	973.8	1159.5	-37.0	1570.6	1334.0	1833.8	1087.3	912.2	1278.9	-30.8	1543.5	1325.6	1777.1	1056.7	880.9	1250.5	-31.5	1685.1	1444.3	1940.7	1059.9	889.5	1240.2	-37.1	1335.1	1137.7	1551.6	869.0	730.3	1046.7	-34.9	1675.8	1417.6	1939.0	1285.5	1089.3	1527.5	-23.3
	65 to 69	2301.2	2157.6	2447.9	1523.3	1398.6	1642.2	-33.8	2121.1	1817.3	2461.4	1614.8	1366.4	1893.6	-23.9	2158.7	1855.2	2522.0	1574.5	1309.9	1836.3	-27.1	2282.8	1966.1	2622.4	1495.5	1269.7	1749.6	-34.5	1851.8	1574.4	2142.0	1265.9	1061.1	1499.9	-31.6	2416.6	2081.7	2817.7	1844.0	1537.9	2177.5	-23.7
	70 to 74	3921.9	3714.6	4155.0	2632.8	2450.2	2830.3	-32.9	3566.9	3074.7	4080.5	2738.3	2342.4	3182.8	-23.2	3768.5	3291.2	4304.1	2850.1	2425.9	3319.5	-24.4	3764.2	3262.8	4298.4	2468.9	2120.3	2865.8	-34.4	3363.9	2880.3	3871.1	2158.7	1839.4	2540.3	-35.8	4241.1	3646.7	4888.4	3323.6	2837.1	3824.1	-21.6
	75 to 79	5003.5	4743.6	5284.3	3660.3	3412.9	3908.3	-26.8	4608.6	4024.2	5248.1	3681.6	3190.0	4226.2	-20.1	5159.5	4530.7	5841.9	4068.6	3510.0	4670.0	-21.1	4844.2	4263.5	5501.8	3367.1	2890.0	3860.2	-30.5	3990.4	3456.8	4575.6	2945.0	2541.0	3421.4	-26.2	5132.7	4541.4	5766.0	4724.8	4073.5	5390.0	-7.9
	80 plus	8154.4	7852.8	8499.2	6191.5	5873.0	6525.6	-24.1	6830.9	6280.5	7427.6	5658.3	5170.0	6143.1	-17.2	9846.5	9039.0	10647.5	7234.9	6608.5	7882.7	-26.5	8508.5	7873.4	9176.3	5808.6	5307.1	6344.6	-31.7	7375.7	6788.1	8034.4	5213.6	4749.2	5749.1	-29.3	7789.7	7193.2	8362.6	7170.1	6553.5	7790.0	-8.0
Low back pain	60 to 64	2305.1	1372.5	3483.3	2286.9	1362.7	3473.4	-0.8	2293.1	1388.0	3472.5	2282.7	1364.3	3486.9	-0.5	2352.6	1467.1	3533.2	2305.7	1394.7	3504.3	-2.0	2323.2	1393.2	3505.4	2312.3	1392.7	3483.8	-0.5	2318.3	1402.3	3459.1	2301.2	1397.4	3539.2	-0.7	2273.5	1358.3	3439.8	2262.7	1353.0	3486.4	-0.5
	65 to 69	2331.3	1479.7	3459.1	2353.9	1483.6	3529.2	1.0	2330.1	1465.9	3513.2	2345.4	1489.3	3528.6	0.7	2368.6	1538.5	3507.9	2374.4	1495.5	3484.2	0.2	2350.8	1485.4	3514.2	2381.1	1499.9	3569.9	1.3	2342.3	1477.3	3467.0	2363.8	1484.9	3543.5	0.9	2293.9	1450.4	3439.0	2332.8	1469.2	3525.2	1.7
	70 to 74	2330.1	1454.2	3482.7	2352.2	1454.2	3512.1	0.9	2317.8	1434.1	3501.6	2342.4	1475.0	3441.3	1.1	2396.5	1514.5	3529.4	2397.2	1488.2	3512.9	0.0	2356.3	1443.9	3560.8	2383.1	1460.9	3598.1	1.1	2313.8	1426.7	3498.3	2356.0	1461.5	3551.2	1.8	2293.0	1425.1	3407.6	2321.4	1431.3	3475.5	1.2
	75 to 79	2346.3	1476.1	3389.2	2361.1	1488.6	3429.0	0.6	2331.5	1451.0	3410.0	2355.5	1471.1	3436.2	1.0	2413.5	1554.9	3485.6	2405.3	1519.9	3528.6	-0.3	2379.4	1488.9	3420.6	2382.5	1500.6	3501.2	0.1	2331.4	1468.4	3405.2	2363.6	1503.8	3412.0	1.4	2309.8	1445.1	3349.1	2335.1	1457.4	3398.2	1.1
	80 plus	2214.5	1495.7	3111.7	2202.3	1501.7	3074.3	-0.6	2170.4	1490.7	3026.2	2175.4	1479.7	3049.2	0.2	2312.8	1562.0	3238.5	2273.4	1538.1	3149.7	-1.7	2271.3	1517.0	3206.1	2239.7	1517.9	3135.5	-1.4	2209.7	1472.0	3117.6	2200.6	1486.5	3104.0	-0.4	2153.1	1473.0	2986.0	2155.9	1466.4	2997.8	0.1
Migraine	60 to 64	762.6	481.7	1124.1	775.3	491.0	1136.6	1.7	774.9	497.7	1142.1	784.1	496.7	1153.8	1.2	767.0	482.6	1118.6	777.0	489.5	1131.9	1.3	725.5	452.4	1111.1	749.0	461.2	1139.9	3.2	757.4	484.7	1120.7	775.3	495.5	1136.9	2.4	755.2	477.0	1129.6	765.2	479.5	1121.8	1.3
	65 to 69	618.0	396.1	906.9	623.6	395.6	907.1	0.9	628.1	401.5	915.7	629.6	398.4	925.2	0.3	626.0	402.3	905.7	626.7	395.7	917.4	0.1	590.7	367.9	901.0	602.7	373.5	912.5	2.0	612.3	393.0	897.8	623.5	399.0	911.1	1.8	609.6	388.0	893.1	614.0	390.5	895.7	0.7
	70 to 74	481.7	306.0	723.4	486.8	312.7	733.2	1.1	486.8	309.1	728.0	490.6	317.1	730.6	0.8	491.0	316.9	716.5	494.2	322.5	736.9	0.7	462.3	287.0	727.5	472.8	294.9	737.0	2.3	473.5	298.3	706.6	483.2	312.2	716.2	2.0	474.9	302.6	709.6	477.8	299.1	715.3	0.6
	75 to 79	370.5	233.9	558.6	376.4	237.6	570.5	1.6	371.5	231.5	556.9	380.4	239.3	571.5	2.4	382.2	243.9	574.4	381.4	245.7	570.3	-0.2	357.2	213.0	554.4	364.6	215.4	564.2	2.1	363.1	227.5	546.5	371.9	232.2	555.2	2.4	365.4	228.0	546.3	371.5	235.7	562.7	1.7
	80 plus	297.1	186.8	446.4	301.3	187.9	444.8	1.4	294.6	186.3	431.7	300.9	189.7	441.9	2.1	308.8	196.5	458.7	310.1	197.7	455.9	0.4	291.1	176.1	449.3	296.0	180.9	456.5	1.7	291.2	183.2	431.3	296.6	185.2	442.3	1.9	294.4	187.3	435.7	296.5	184.6	440.4	0.7
Neck pain	60 to 64	534.2	326.0	828.6	527.1	323.1	808.0	-1.3	554.7	337.2	856.1	549.8	338.9	837.4	-0.9	552.9	335.9	854.2	546.6	339.5	843.1	-1.1	465.5	273.0	725.4	458.2	268.5	707.0	-1.6	552.0	328.1	852.3	547.2	337.2	851.4	-0.9	552.1	333.9	851.2	545.8	336.4	839.9	-1.1
	65 to 69	642.4	389.0	1007.4	637.6	388.3	989.0	-0.7	668.4	404.5	1033.3	663.5	407.3	1034.6	-0.7	665.4	403.4	1023.9	661.0	396.0	1027.4	-0.7	561.8	322.6	896.0	555.7	315.3	882.3	-1.1	664.4	395.7	1030.5	661.6	400.8	1015.7	-0.4	662.7	407.9	1022.6	658.3	400.1	1010.5	-0.7
	70 to 74	683.5	431.4	1065.2	679.8	428.4	1042.9	-0.5	711.3	449.2	1102.8	709.1	445.9	1080.3	-0.3	709.5	449.7	1105.6	707.6	451.6	1091.8	-0.3	599.3	359.0	955.6	587.0	349.8	935.2	-2.0	703.6	440.7	1087.6	704.6	439.5	1067.6	0.2	705.6	443.2	1093.7	702.7	443.8	1074.0	-0.4
	75 to 79	631.2	389.0	997.7	627.1	385.5	985.0	-0.6	655.3	403.9	1036.1	653.7	403.7	1032.6	-0.3	656.1	409.4	1024.5	650.7	401.5	1022.4	-0.8	548.5	317.9	910.6	543.3	315.3	898.5	-1.0	650.3	407.0	1020.6	649.5	399.6	1019.5	-0.1	652.2	399.5	1014.6	650.5	396.4	1025.6	-0.3
	80 plus	614.8	398.8	918.3	602.1	394.7	886.6	-2.1	622.9	412.8	909.4	615.6	411.4	890.5	-1.2	639.9	416.5	941.9	624.5	412.3	923.7	-2.4	547.6	337.6	855.9	531.5	334.2	816.0	-2.9	637.7	413.3	956.1	622.9	409.9	912.5	-2.3	623.1	410.9	907.5	617.3	410.8	898.5	-0.9
Osteoarthritis	60 to 64	341.0	168.1	679.3	374.7	183.2	748.0	9.9	324.6	160.2	646.8	356.1	175.1	713.0	9.7	349.3	171.9	698.6	377.2	184.2	758.5	8.0	356.7	177.7	711.3	391.7	189.1	774.8	9.8	335.3	164.4	659.3	366.3	178.1	731.5	9.2	325.6	159.2	648.7	358.7	174.0	724.0	10.2
	65 to 69	418.8	204.1	833.3	457.9	225.7	908.4	9.3	399.6	197.7	793.8	435.4	214.1	860.6	9.0	428.8	210.9	849.9	461.7	228.6	918.0	7.7	437.6	211.3	863.5	477.2	236.2	947.3	9.0	411.6	202.7	825.0	448.6	216.0	900.2	9.0	400.0	197.7	798.6	438.8	215.3	887.2	9.7
	70 to 74	490.8	246.4	984.5	532.7	266.8	1059.5	8.6	470.2	238.1	958.2	509.7	251.4	1025.0	8.4	501.4	249.0	1007.5	536.7	265.5	1065.4	7.0	511.3	250.4	1030.8	554.3	273.7	1096.5	8.4	481.3	238.8	969.3	522.7	259.0	1065.9	8.6	470.0	234.9	954.1	512.2	258.5	1014.3	9.0
	75 to 79	548.6	277.9	1109.6	594.0	301.0	1192.2	8.3	527.3	266.1	1073.8	569.2	286.8	1154.5	7.9	560.5	283.0	1121.2	597.8	301.9	1192.9	6.6	571.8	285.5	1162.3	617.2	314.2	1224.3	7.9	539.2	269.6	1086.9	584.0	291.7	1169.9	8.3	527.9	269.3	1073.4	574.5	291.0	1164.5	8.8
	80 plus	612.1	311.3	1219.8	664.3	339.9	1326.6	8.5	598.2	305.0	1214.3	645.4	330.8	1295.6	7.9	623.8	315.5	1251.1	669.4	346.0	1328.9	7.3	634.7	321.2	1257.1	687.5	348.8	1351.1	8.3	598.5	306.4	1187.0	650.9	331.8	1305.6	8.8	596.9	305.2	1184.5	646.0	333.3	1289.8	8.2
Alzheimer's disease and other dementias	60 to 64	112.8	70.5	166.2	94.8	59.2	140.3	-16.0	106.2	65.7	163.3	87.0	53.0	130.9	-18.0	112.2	68.1	170.0	94.9	58.3	142.2	-15.4	128.4	80.0	188.9	109.0	67.0	161.8	-15.1	107.3	62.9	164.0	88.8	54.2	134.5	-17.2	106.2	67.4	162.5	87.7	53.2	131.3	-17.4
	65 to 69	259.1	168.4	388.9	217.0	141.1	327.3	-16.2	245.5	157.4	371.5	199.8	126.2	300.2	-18.6	261.2	161.1	397.9	217.6	137.2	331.9	-16.7	285.4	183.2	416.1	248.1	159.3	379.5	-13.1	249.1	157.8	376.1	205.0	130.2	310.5	-17.7	247.0	157.4	377.8	200.6	126.0	303.4	-18.8
	70 to 74	609.7	401.3	863.3	514.0	335.8	733.0	-15.7	587.1	386.3	847.2	475.8	314.4	687.1	-19.0	622.1	407.7	895.1	518.5	340.8	743.4	-16.7	643.4	429.5	894.8	579.5	382.7	831.8	-9.9	592.1	394.0	846.5	487.7	321.0	695.9	-17.6	587.9	386.8	843.5	479.8	317.5	687.3	-18.4
	75 to 79	1108.6	745.1	1586.6	931.7	617.6	1327.7	-16.0	1076.3	714.3	1545.4	867.6	575.3	1232.8	-19.4	1140.4	766.3	1657.1	944.9	625.9	1357.4	-17.1	1146.4	759.5	1592.4	1033.0	696.1	1464.7	-9.9	1083.0	717.4	1561.7	891.6	590.8	1268.5	-17.7	1075.1	706.4	1562.9	875.7	582.5	1272.3	-18.5
	80 plus	2957.7	2037.3	4008.3	2558.3	1759.1	3486.8	-13.5	3072.2	2119.2	4186.5	2543.3	1735.9	3451.4	-17.2	2949.5	2052.4	4012.7	2605.1	1802.6	3548.3	-11.7	2868.7	1989.2	3871.4	2663.3	1842.0	3610.7	-7.2	2711.2	1851.8	3696.3	2427.2	1670.1	3315.0	-10.5	3026.8	2084.3	4111.4	2486.6	1711.3	3382.6	-17.8

## Discussion

In Brazil, there is a consistent decrease in age-standardized DALY rates, as already described [6]. These results, as well as the increase in life expectancy, are probably the results of improving social conditions, broader access to health care aside to the priority given to prevention and basic care [6, 17]. However, the increase in the number and proportion of DALYs among elderlies suggests this age group presents a higher burden of disease. In addition, premature death is the main component of the burden of disease for Brazilian older adults, leading to a 10-year gap between life expectancy in Brazil and the high-income countries [10]. Regional inequality persists; the states with lower SDI from the Northeast and North regions still present higher mortality rates.

The smaller increase of HALE than life expectancy in the last 16 years means that, despite living more, people spend a substantial time of their old age with disability and illness. This burden of disease may imply a restriction on the ability to contribute to the labor force at old age. This subject is a crucial aspect currently under discussion, with the Congress intent to extend the age bracket for retirement and pension receiving [18].

The higher proportion of NCDs as a cause of death and disability among the older adults is expected, as well as the increase of the burden of disease with aging. However, it is noteworthy that the leading causes of death are mostly preventable NCDs: ischemic heart disease, ischemic and hemorrhagic stroke, chronic obstructive pulmonary disease, diabetes, and breast, lung, and stomach cancers. The social inequality may be responsible for the increasing trends of prostate cancer in Northeast and North regions and the increase of death by diabetes in the North region, the older adults with lower access to health prevention [17].

Many of these preventable causes of death require an approach of risk factors prevention and management throughout the life course to lower their rates. It is thus fundamental to strengthen the national health promotion policy implemented in 2006, and to articulate the various public actions to stimulate healthy eating and regular practice of physical activity, to prevent and control smoking, and to reduce morbidity and mortality due to alcohol and other drug abuse [19]. In order to guarantee a good control of the NCD in a socially deprived country, it is advisable for Brazil to maintain the investments on primary health care and the free access to medications for hypertension, diabetes, and smoking cessation implemented in the country since 1998 [20].

We hypothesized the decrease in COPD and the corresponding increase in lower respiratory infections; mortality rates may represent a shift in the way doctors fill out the death certificates, the underlying cause of death being more poorly specified among older adults, the age group that usually concentrates the highest rates of garbage codes [21].

The burden for years lived with disability reveals important causes of personal and family suffering, such as vision and hearing impairments, musculoskeletal pain, and mood disorders. A recent study showed that lower back pain is a disabling condition, associated with psychological factors, lower education, and income level in Brazil [22]. Musculoskeletal diseases are four of the top 10 leading causes of disability, suggesting the need for investments in the areas of prevention and rehabilitation.

Depressive disorders generally present a minor burden to older adults than to the younger ones [23]. Social determinants may explain the higher burden of depression among Brazilian older adult women. Current Brazilian elderly are composed predominantly of women with low education and unpaid work during adulthood, and who experience greater chances of widowhood and disadvantageous socioeconomic status. Nevertheless, if the policies of universalization of social security persist, with the guarantee of pensions for housewives, they tend to become heads of families and providers, with greater participation in extra-community activities and socialization than men [24].

The aging of the population accounts for much of the increased diabetes burden in Brazil, and efforts to control the epidemic of obesity and physical inactivity must continue [25, 26].

Accidental falls significantly increase the risk of disability, and the present study supports this fact. Aside from physical damage, a fall can lead to psychological consequences, such as fear of falling. Fear of falls is associated with several health problems, such as restriction or limitation of activities, loss of muscle strength and postural control, a negative perception of health, depression, and social isolation [27].

The increase in the burden of Alzheimer’s disease and other dementias imposes an important public health issue regarding investments in social and medical care [28]. Although age-standardized mortality did not increase, the prevalence increased five times in the world, mainly because of aging population. With limited scope for prevention and the absence of an effective disease-modifying treatment, the burden on family, caregivers, and health care system will continue to increase rapidly [29].

## Strength and limitations

The GBD study has by strength to correct the mortality data and the standardization of metrics, allowing a subnational analysis in Brazil. In addition, the IHME website provides all metrics by gender and age, in addition to age-standardized rates. All sources of information and analyses are available on the IHME website as well in the appendix of the published papers.

However, a lack of primary data and problems in data quality on the subnational level may limit its analysis, the estimates from the most developed regions being more reliable. The higher life expectancy at birth and at 60 years in the states with the lowest SDI may represent the poorer quality of data in the less developed North and Northeast regions, as well as the occurrence of problems in the IBGE estimates of the elderly population. A lack of information for non-fatal diseases may explain the large 95% UI for YLD estimates.

## Conclusions

The burden of disease is shifting toward the elderly in Brazil. Greater longevity is moving the population to a condition of increased morbidity and disability. Diseases that are sensitive to prevention and control throughout the lifespan comprise most of the burden of disease among the older Brazilian adults, in an ambient of social health inequalities. Health policies must face these challenges so the Brazilian elderly may achieve longevity with quality of life.

## Data Availability

The data we used in this article are publicly available online on the official website of the Institute of Health Metrics and Evaluation (http://ghdx.healthdata.org/gbd-results-tool).

## Abbreviations

CW region: Central-West region
DALY: Disability-adjusted life year
GBD: Global Burden of Disease
HALE: Healthy life expectancy
SIH: Hospital Information System
SINAN: Injury Information System
IBGE: Institute of Geography and Statistics
IHME: Institute of Health Metrics and Evaluation
LE: Life expectancy at birth
SIM: Mortality Information System
NCD: Non-communicable diseases
N region: North region
NE region: Northeast region
SIA: Outpatient Information System
SDI: Socio-demographic index
S region: South region
SE region: Southeast region
YLD: Years lived with disability
YLL: Years of life lost
95% UI: 95% uncertainty intervals
